# Prophylactic, Synthetic Intraperitoneal Mesh Versus No Mesh Implantation in Patients with Fascial Dehiscence

**DOI:** 10.1007/s11605-018-3873-z

**Published:** 2018-07-23

**Authors:** Manuel O. Jakob, Daniel Spari, Joel Zindel, Tawan Pinworasarn, Daniel Candinas, Guido Beldi

**Affiliations:** Department of Visceral Surgery and Medicine, Bern University Hospital, Bern, Switzerland

**Keywords:** Fascial dehiscence, Non-absorbable mesh, Polypropylene mesh, Polyester mesh

## Abstract

**Background:**

Primary closure of post-operative facial dehiscence (FD) is associated with a high incidence of recurrence, revisional surgery, and incisional hernia. This retrospective study compares outcomes of implantation of non-absorbable intra-abdominal meshes with primary closure of FD. The outcomes of different mesh materials were assessed in subgroup analysis.

**Methods:**

A total of 119 consecutive patients with FD were operated (70 mesh group and 49 no mesh group) between 2001 and 2015. Primary outcome parameter was hernia-free survival. Secondary outcome parameters include re-operations of the abdominal wall, intestinal fistula, surgical site infections (SSI), and mortality. Kaplan-Meier analysis for hernia-free survival, adjusted Poisson regression analysis for re-operations and adjusted regression analysis for chronic SSI was performed.

**Results:**

Hernia-free survival was significantly higher in the mesh group compared to the no mesh group (*P* = 0.005). Fewer re-operations were necessary in the mesh group compared to the no mesh group (adjusted incidence risk ratio 0.44, 95% confidence interval [CI] 0.20–0.93, *P* = 0.032). No difference in SSI, intestinal fistula, and mortality was observed between groups. Chronic SSI was observed in 7 (10%) patients in the mesh group (*n* = 3 [6.7%] with polypropylene mesh and 4 [28.6%] with polyester mesh). The risk for chronic SSI was significantly higher if a polyester mesh was used when compared to a polypropylene mesh (adjusted odds ratio 8.69, 95% CI 1.30–58.05, *P* = 0.026).

**Conclusion:**

Implantation of a polypropylene but not polyester-based mesh in patients with FD decreases incisional hernia with a low rate of mesh-related morbidity.

**Electronic supplementary material:**

The online version of this article (10.1007/s11605-018-3873-z) contains supplementary material, which is available to authorized users.

## Introduction

Fascial dehiscence (FD) is a surgical complication in which the fascia dissociates along the surgical incision and potentially leads to evisceration. The incidence ranges from 0.5 to 2.9% following abdominal surgery.^1^,^2^ Potential risk factors for FD include technical failure, elevated intra-abdominal pressure (e.g., in obese patients), and deep or superficial surgical site infection (SSI).^3^

Patients after FD are at high risk of re-rupture of the abdominal fascia following primary closure with an incidence of up to 44.4%.^4^ This recurrence might potentially end in an open abdomen requiring multiple revisional operations. Additionally, these patients are at exceedingly high risk of developing incisional hernia with an incidence of up to 83% of patients.^5^

Despite such high rates of complications, little evidence-based therapeutic or prophylactic options are currently available. Strategies to treat FD include (1) direct closure of the abdominal wall, (2) implantation of an absorbable mesh, and (3) implantation of a non-absorbable mesh. Because the first option is associated with a high incidence of recurrent FD and incisional hernia,^4^,^6^ implantation of meshes has been attempted to prevent recurrent FD.^7^ However, usage of absorbable meshes did not reduce incisional hernia in comparison to direct closure of the abdominal wall.^8^

Thus, only implantation of non-absorbable meshes potentially prevents both, early recurrence of FD and incisional hernia formation.^6^,^9^ Despite such theoretical advantages, prosthetic non-absorbable meshes are not commonly used mainly because of potential risks of mesh-related morbidity in a contaminated or dirty surgical field, which is typically found in patients with FD.^10^ Such restrictive use, however, is not based on solid scientific data beyond case reports. While some evidence exists showing safety in hernia surgery in the contaminated field, evidence is lacking of the use of non-absorbable meshes in FD.^11^,^12^ Such analysis is complicated by the plethora of meshes currently available. Mesh material potentially determines survival and clearance of bacteria as well as biofilm formation.^13^,^14^ In particular, multi-filamentous polyester meshes (PE) seem to be more prone to chronic SSI because of decreased bacterial resistance and clearance compared to polypropylene meshes (PP).^15^

This study is based on the hypothesis that non-absorbable meshes may reduce recurrent FD and incisional hernia. Thus, the aim of this single-center study was to compare the results of non-absorbable mesh implantation with conventional closure using a single running suture in patients with FD. The goal was to obtain a risk-benefit assessment of the potential treatment strategies in this severe clinical condition. In order to determine the relevance of mesh material, a subgroup analysis was performed to compare between PP and PE meshes.

## Materials and Methods

### Study Design

In this retrospective study, all medical records of patients treated for FD between January 2001 and December 2015 were screened. In total, 171 patients with FD were identified. Then, 52 patients were excluded because no primary fascial closure was achieved (laparostomy or open abdomen) resulting in 119 patients analyzed in the current study. This study has been approved by the ethics committee of Bern, Switzerland.

Patients who underwent intraperitoneal mesh implantation (mesh group) were compared to primary fascial closure (no mesh group). In 2006, intraperitoneal, non-absorbable mesh implantation became standard of care at the institution for the treatment of patients with FD because of the clinical observation of severe abdominal wall complications in patients with primary fascial closure (recurrent FD, hernia). Thus, assignment to groups was dependent on the year of treatment: 79.6% (39/49) of patients in the no mesh group were treated before 2006 and 20.4% (10/49) were treated after 2006. Reasons for no mesh treatment after 2006, thus deviation from standard of care (*n* = 10), were severe intra-abdominal contamination or underlying disease (supporting information, Table S1).

Primary outcome parameter was hernia-free survival. Follow-up was recorded at 3 months, 1 year, and 2 years in addition to the last follow-up. Hernia-free was defined as no occurrence of a clinically apparent hernia. Clinically apparent hernia was defined as (1) if an operation was performed because of hernia or (2) if clinically identified by an independent observer. Secondary outcome parameters comprised mesh-related morbidity such as intestinal fistula, SSI, partial mesh explantation, and re-operations. Different mesh materials (polypropylene (PP), polyester (PE)) were assessed in conjunction to mesh-related morbidity.

Post-surgical complications were classified according to Clavien-Dindo^16^ and SSI according to CDC.^17^ Chronic SSI was defined by clinical evidence of SSI lasting longer than 268 days, which represents the median duration of SSI in the mesh group. Intestinal (enterocutaneous) fistula was defined as cutaneous secretion of bowel contents after FD repair. Re-operations were defined as revisional surgeries under general anesthesia related to complications of the abdominal wall. Revisional surgeries included fistula takedown, revisional surgery because of SSI, incisional hernia, reconstruction of the abdominal wall, and partial or complete mesh explantation. Partial mesh explantation was defined as a surgical procedure removing parts of the mesh under general anesthesia. Anastomotic leakage was defined as a defect of the anastomotic site associated with a leak of intestinal contents. The Mannheim peritonitis index was assessed using the previously published weighting score.^18^ The grade of contamination and existing adhesions was assessed using the Björck classification.^19^

In addition to our own historical cohort (no mesh implantation), published series with and without non-absorbable mesh implantation were identified and important outcome parameter (incisional hernia, intestinal fistula, SSI, mesh explantation) were recorded and compared to the current results. The results were plotted with the outcome parameter on the *Y*-axis and duration of follow-up on the *X*-axis.

### Surgical Technique

Patients with FD were taken to the operating room. First, the underlying pathology was explored and treated adhering to standards. The abdominal cavity was extensively rinsed with saline.

In the no mesh group, the abdominal fascia was primarily closed with a single running suture using loop 0-PDS (PDS®, Ethicon Sarl) in a standardized technique using a suture:wound ratio of 1:4. The skin was closed using loose single sutures or a vacuum dressing.

In the mesh group, a non-absorbable mesh was implanted intraperitoneally before fascial closure. The chosen mesh material (PP vs. PE) was at the discretion of the responsible surgeon. The meshes overlapped the incision by at least 5 cm on all sides. Any form of preperitoneal dissection has been avoided. The mesh was fixed using running 3–0 polypropylene sutures interrupted every 10 cm (Prolene®, Ethicon Sarl). The fascia was closed using a 0 PDS running suture (PDS®, Ethicon Sarl) in a standardized technique using a suture:wound ratio of 1:4. The skin was closed using loose single sutures or a vacuum dressing. The application of a vacuum dressing was at the discretion of the surgeon, e.g., if a SSI was highly likely to occur. The dressings were changed at an interval of 3–4 days. The vacuum dressings were removed when there was evidence of granulation tissue in addition to clean wound conditions.

### Statistics

Data are reported as median and interquartile ranges (IQR), or numbers and percentages. The primary endpoint, hernia-free survival, was analyzed with Kaplan-Meier curves and a log-rank test for statistical comparison. Secondary outcomes were compared using Fisher’s exact test and Mann-Whitney *U* test for categorical and continuous variables respectively. Effects are reported as risk differences or c-statistics with 95% confidence intervals (CI). The effect of mesh treatment on hernia-free survival and secondary outcomes was further tested in a univariable and multivariable (adjusted) regression model. Clinically important variables (age, gender, BMI, smoker, malignancy, ASA score, immunosuppressive drugs, and type of laparotomy) were tested and included in the regression model. Co-variables with a *P* value below 0.2 on univariable analysis were included. Logistic, Cox, or Poisson regression were fitted for binary, time-to-event, or count outcomes, respectively. Results are reported as odds ratio (OR), hazard ratio (HR), or incidence risk ratio (IRR) with 95% CI and *P* values. Identified studies reporting synthetic mesh vs no mesh in patients with fascial dehiscence were summarized using the random effects model. All statistical tests were performed by using SPSS Statistics (Version 17.0.0, SPSS Inc., Chicago, IL). A two-sided *P* value of < 0.05 was considered statistically significant.

## Results

Different meshes used in our patient cohorts were in 54 (77.1%) patients a large-pore, mono-filamentous, dual-layered PP mesh [in 50 (71.4%) patients Parietene Composite®, Medtronic, and in 4 (5.7%) patients Dynamesh®, FEG Textiltechnik mbH] and in 16 (22.9%) patients a large-pore, multi-filamentous, dual-layered PE mesh (Parietex Composite®, Medtronic).

### Short-Term Outcome

Patient characteristics are shown in Table 1. Detailed operative characteristics are shown in Table 2. During surgery for FD, there was no difference in anastomotic leakage between the two groups. Post-operative results are shown in Table 3. There was no statistically significant difference in intestinal fistula and in-hospital mortality between the two groups. A significant decrease in complications above grade IIIa according to Clavien-Dindo was found if a mesh was implanted (*P* = 0.034) leading to an adjusted odds ratio (OR) of 0.46 (*P* = 0.067, Table 4).

**Table 1:** Patient’ characteristics

	No mesh (*n* = 49)	Mesh (*n* = 70)	*P* value†
Median age, years (IQR)	64 (56–74)	66 (58–74)	0.389‡
Male patients, *n* (%)	32 (65.3%)	45 (64.3%)	1.000
Median preoperative body mass index, kg/m^2^ (IQR)	28.1 (23.6–34.9)	25.8 (22.9–29.1)	0.110‡
Active smoker, *n* (%)	12 (24.5%)	21 (30.0%)	0.835
Former smoker, *n* (%)	9 (18.4%)	10 (14.3%)	0.439
Non-smoker, *n* (%)	22 (44.9%)	38 (54.3%)	0.846
Known malignancy, *n* (%)	16 (32.7%)	40 (57.1%)	0.010
Diabetes type 1, *n* (%)	–	1(1.4%)	1.000
Diabetes type 2, *n* (%)	6 (12.2%)	11 (15.7%)	0.791
Cardiac disease, *n* (%)	15 (30.6%)	34 (48.6%)	0.125
Pulmonary disease, *n* (%)	14 (28.6%)	29 (41.4%)	0.325
Liver disease, *n* (%)	13 (26.5%)	16 (22.9%)	0.516
Kidney disease, *n* (%)	12 (24.5%)	17 (24.3%)	0.829
Anticoagulation, *n* (%)			
Phenprocoumon	4 (8.2%)	8 (11.4%)	0.762
Heparin	–	2 (2.9%)	0.519
Platelet aggregation inhibitors	9 (18.4%)	19 (27.1%)	0.505
Dual anticoagulation	–	1 (1.4%)	1.000
Immunosuppressors, *n* (%)			
Immunosuppressive drugs	3 (6.1%)	9 (12.9%)	0.354
Cortisone	4 (8.2%)	8 (11.4%)	0.759
Both	1 (2.0%)	–	0.415
ASA score, *n* (%)			
1	–	1 (1.4%)	0.837
2	6 (12.2%)	9 (12.9%)	
3	30 (61.2%)	44 (62.9%)	
4	7 (14.3%)	13 (18.6%)	
Emergency primary procedure, *n* (%)	27 (55.1%)	34 (48.6%)	0.578
Number of prior laparotomies (IQR)	1 (1–2)	2 (1–1)	0.038‡
Duration of primary operation, minutes (IQR)	223 (164–319)	229 (180–330)	0.844‡
Incision at primary procedure, *n* (%)			
Median laparotomy	38 (77.6%)	55 (78.6%)	0.477
Transverse laparotomy	6 (12.2%)	12 (17.1%)	0.793
Combined	1 (2.0%)	3 (4.3%)	1.000
Type of primary procedure, *n* (%)			
Cholecystectomy	4 (8.2%)	1 (1.4%)	0.053
Hepatobiliary surgery	4 (8.2%)	5 (7.1%)	
Pancreatic surgery	1 (2.0%)	2 (2.9%)	
Upper GI surgery	4 (8.2%)	6 (8.6%)	
Resection of intestine with preserved continuity	20 (40.8%)	20 (28.6%)	
Resection of intestine without preserved continuity	12 (24.5%)	17 (24.3%)	
Multi-visceral resections	–	9 (12.9%)	
Nephrectomy/kidney transplantation	1 (2.0%)	4 (5.7%)	
Other*	3^a^ (6.1%)	6^b^ (8.6%)	

*Others included ^a^1 aortic aneurysms, 1 appendectomy, 1 Fournier gangrene, ^b^1 cesarean section, 2 aortic aneurysms, 2 staging laparotomies, 1 abdominal flap

†Fisher exact test, except ‡Mann-Whitney *U* test

**Table 2:** Operative characteristics during treatment of FD and post-operative results

	No mesh (*n* = 49)	Mesh (*n* = 70)	Effect measure (95% CI)*	*P* value†
Occurrence of FD after primary surgery, days (IQR)	9 (7–16)	12 (8–16)	0.6 (0.4–0.7)	0.249‡
Leakage of small intestine, *n* (%)	4 (8.2%)	5 (7.1%)	− 1.0 (− 10.8–8.7)	1.000
Leakage of colonic anastomosis, *n* (%)	9 (18.4%)	7 (10%)	− 8.4 (− 21.3–4.6)	0.274
Pancreatic fistula, *n* (%)	–	2 (2.9%)	2.9 (− 1.1–6.8)	0.511
Combination of small intestinal and colonic anastomosis leakage, *n* (%)	1 (2.0%)	1 (1.4%)	− 0.6 (− 5.4–4.2)	1.000
Mannheim peritonitis index, *n* (IQR)	15 (9–23)	12 (9–25)	0.5 (0.1–0.4)	0.870‡
Björk classification at FD, *n* (%)				
1A: clean without adhesion	3 (6.8%)	2 (3.0%)	− 3.8 (− 12.3–4.7)	0.032
1B: contaminated without adhesion	8 (18.2%)	19 (28.8%)	10.6 (− 5.2–26.4)	
2A: clean with beginning adhesion formation	–	7 (10.6%)	10.6 (3.2–18.0)	
2B: contaminated with beginning adhesion formation	31 (70.4%)	35 (53.0%)	− 17.4 (− 35.5–0.7)	
3: intestinal fistula	2 (4.5%)	3 (4.5%)	0.0 (− 8.0–8.0)	
VAC therapy after FD, *n* (%)	28 (57.1%)	40 (57.1%)	0.0 (− 0.2–18.1)	1.000
Duration of redo-surgery, minutes (IQR)	120 (73–203)	120 (90–160)	0.5 (0.4–0.6)	0.813‡
Patients needing intensive care, *n* (%)	21 (42.9%)	25 (35.7%)	− 7.2 (− 25.0–10.7)	0.450
Days at intensive care unit (IQR)	7 (3–20)	4 (2–9)	0.4 (0.2–0.5)	0.098‡
Number of redo surgeries after FD during hospitalization (IQR)	1 (1–1)	1 (1–1)	0.5 (0.5–0.6)	0.299‡
RBC transfusion necessary, *n* (%)	18 (41.9%)	22 (33.8%)	− 8.0 (− 26.7–10.7)	0.422
RBC transfusion, units (IQR)	2 (1–3)	2 (1–2)	0.4 (0.2–0.6)	0.336‡
Respiratory support, *n* (%)	10 (22.7%)	11 (16.9%)	5.8 (−21.2–9.6)	0.468
Hours of respiratory support (IQR)	72 (37–276)	96 (24–312)	0.5 (0.2–0.7)	0.888‡
Vasoactive drugs after FD, *n* (%)	8 (17.8%)	11 (16.9%)	− 0.9 (− 15.3–13.6)	1.000
Days of support with vasoactive drugs (IQR)	1 (1–1)	1 (1–1)	0.6 (0.4–0.8)	0.240‡
Duration of hospital stay, days (IQR)	24 (18–47)	30 (21–44)	0.6 (0.4–0.7)	0.260‡
Duration of hospital stay after FD, days (IQR)	17 (11–32)	16 (11–28)	0.5 (0.1–0.6)	0.770‡

*FD* fascial dehiscence, *RBC* red blood cell

*Risk difference for categorical and c-statistic for continuous variables. Values in parentheses are 95% confidence intervals. †Fisher exact test, except ‡Mann-Whitney *U* test

**Table 3:** Short-term outcome

	No mesh (*n* = 49)	Mesh (*n* = 70)	Effect measure (95% CI)*	*P* value†
Fistula of small intestine, *n* (%)	5 (10.2%)	4 (5.7%)	− 4.5 (14.6–5.6)	0.485
Colonic fistula, *n* (%)	1 (2.0%)	–	− 2.0 (− 6.0–1.9)	0.412
Pre-existing intestinal fistula at FD, *n* (%)	1 (2.0%)	3 (4.3%)	2.2 (− 3.9–8.4)	0.267
Fistula within 30 after FD, *n* (%)	3 (6.1%)	–	− 6.1 (− 12.8–0.6)	0.067
In-hospital mortality, *n* (%)	9 (18.4%)	11 (15.7%)	2.7 (− 16.5–11.1)	0.805
Reason for mortality, *n* (%)				
Cardiopulmonary insufficiency	4 (8.2%)	2 (2.9%)	5.3 (− 13.9–3.3)	0.161
Sepsis/multi-organ failure	1 (2.0%)	3 (4.3%)	2.3 (− 3.9–8.4)	
Underlying disease	1 (2.0%)	4 (5.7%)	3.7 (− 3.1–10.4)	
Other	3 (6.1%)	2 (2.9%)	− 3.3 (− 11.0–4.5)	
Complications grade > II after FD, *n* (%)	35 (71.4%)	36 (51.4%)	− 20.7 (− 38.0–− 3.5)	0.034
Complications according to Clavien-Dindo after FD (*n* = 48 versus 69) *n* (%)				
0	3 (6.2%)	15 (21.4%)	15.2 (3.4–27.0)	0.174
I	4 (8.3%)	9 (12.9%)	4.5 (− 6.6–15.6)	
II	6 (12.5%)	9 (12.9%)	0.4 (− 11.9–12.6)	
IIIa	8 (16.7%)	5 (7.1%)	− 9.5 (− 21.7–2.6)	
IIIb	4 (8.3%)	5 (7.1%)	− 1.2 (− 11.1–8.7)	
IVa	3 (6.3%)	6 (8.6%)	2.3 (− 7.2–11.8)	
IVb	11 (22.9%)	8 (11.4%)	− 11.5 (− 25.5–2.5)	
V	9 (18.8%)	12 (17.1%)	− 1.6 (15.7–12.5)	

*FD* fascial dehiscence

*Risk difference for categorical and c-statistic for continuous variables. Values in parentheses are 95% confidence intervals. †Fisher exact test

**Table 4:** Regression analyses: mesh vs no mesh in patients with fascial dehiscence

	Unadjusted model		Adjusted model	
Complications >II*^a^	Odds ratio (95% CI)	*P* value	Odds ratio (95% CI)	*P* value
	0.41 (0.18–0.90)	0.026	0.46 (0.21–1.05)	0.067
Hernia-free survival†^b^	Hazard ratio (95% CI)	*P* value	Hazard ratio (95% CI)	*P* value
	0.31 (0.13–0.73)	0.007	0.27 (0.10–0.72)	0.008
Re-operations‡^c^	Incidence risk ratio (95% CI)	*P* value	Incidence risk ratio (95% CI)	*P* value
	0.49 (0.21–1.07)	0.057	0.44 (0.20–0.93)	0.032

Odds ratios, hazard ratios, and incidence risk ratios are shown mesh versus no mesh

*Logistic regression

†Cox-regression

‡Poisson regression

^a^Adjusted for ASA-score

^b^Adjusted for ASA-score, type of laparotomy (median and transverse), immunosuppressants

^c^Adjusted for age, type of laparotomy (median and transverse)

### Long-Term Outcome

**Fig. 1:**
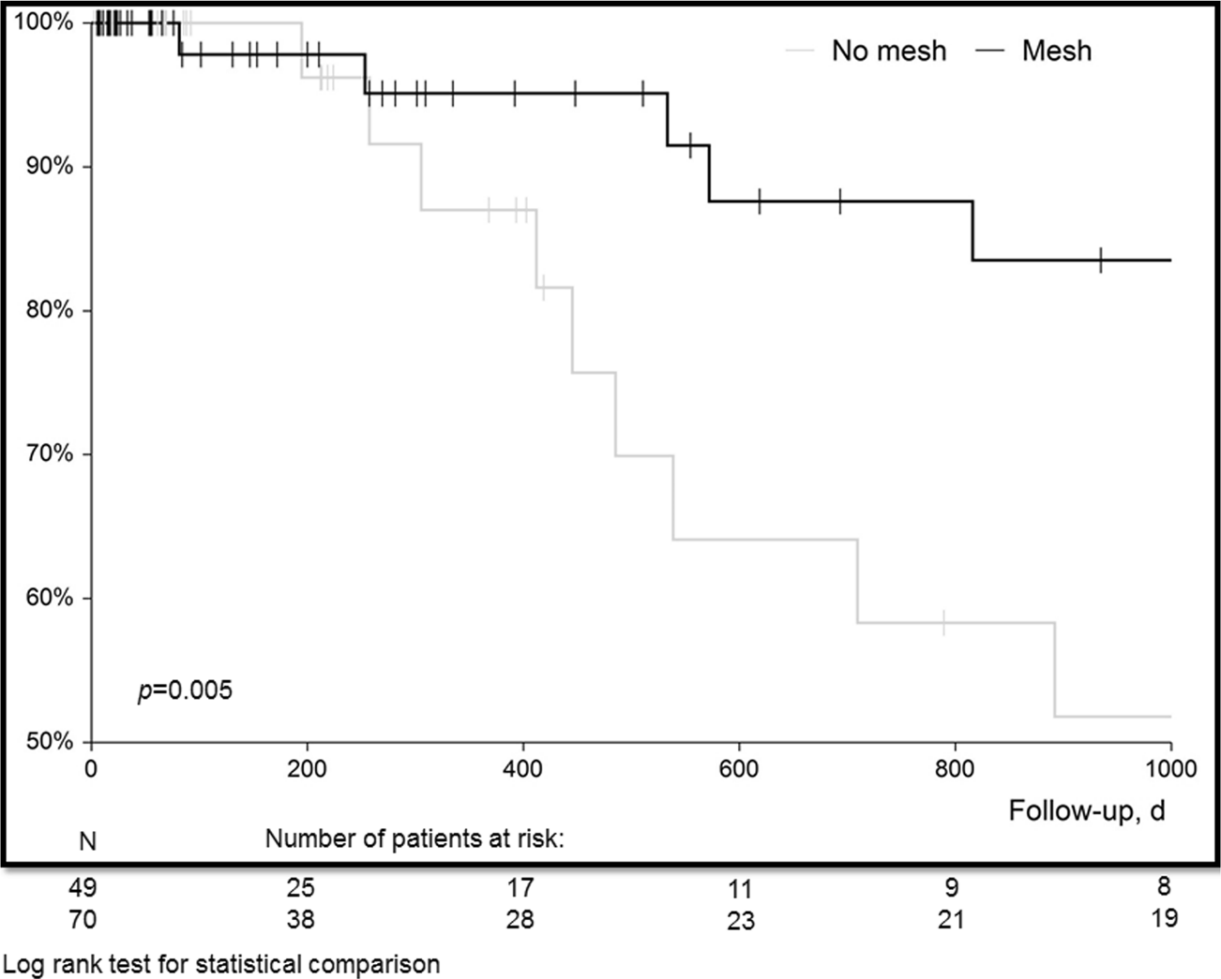
Kaplan-Meier analysis of hernia-free survival

SSI at last follow-up was observed in 12 (30.0%) patients in the no mesh group and in 10 (16.9%) patients in the mesh group (risk difference − 13.1, 95% CI − 30.2–4.1, *P* = 0.145) after a median follow-up of 281 days (IQR 69–770) in the no mesh group and 335 days (IQR 84–1304) in the mesh group (*P* = 0.441). Median rate of re-operations was significantly higher in the no mesh group compared to the mesh group (*P* < 0.001). The incidence of re-operations per 1000 person days was 0.88 (95% CI 0.57–1.31) in the no mesh group and 0.44 (95% CI 0.21–0.80) in the mesh group. Adjusted incidence risk ratio for re-operations (probability of re-operation per person time) for the mesh group vs no mesh group was 0.44 (95% CI 0.20–0.93, *P* = 0.032, Table 4). Incisional hernia was observed in 15 of 40 (37.5%) patients in the no mesh group and 8 of 59 (13.6%) patients in the mesh group (risk difference − 23.9, 95% CI − 41.3 to − 6.6, *P* = 0.008). Hernia-free survival showed a significantly different curve separation when comparing mesh vs no mesh (*P* = 0.005, Fig. 1). The site of hernia recurrence in the mesh group was mainly at the borders of the meshes (*n* = 2) or after mesh removal because of infectious complications in an intention to treat analysis (*n* = 2). Adjusted Cox regression analysis revealed non-absorbable mesh implantation as a significant protective factor for incisional hernia (HR 0.27, 95% CI 0.10–0.72, *P* = 0.008, Table 4).

**Fig. 2:**
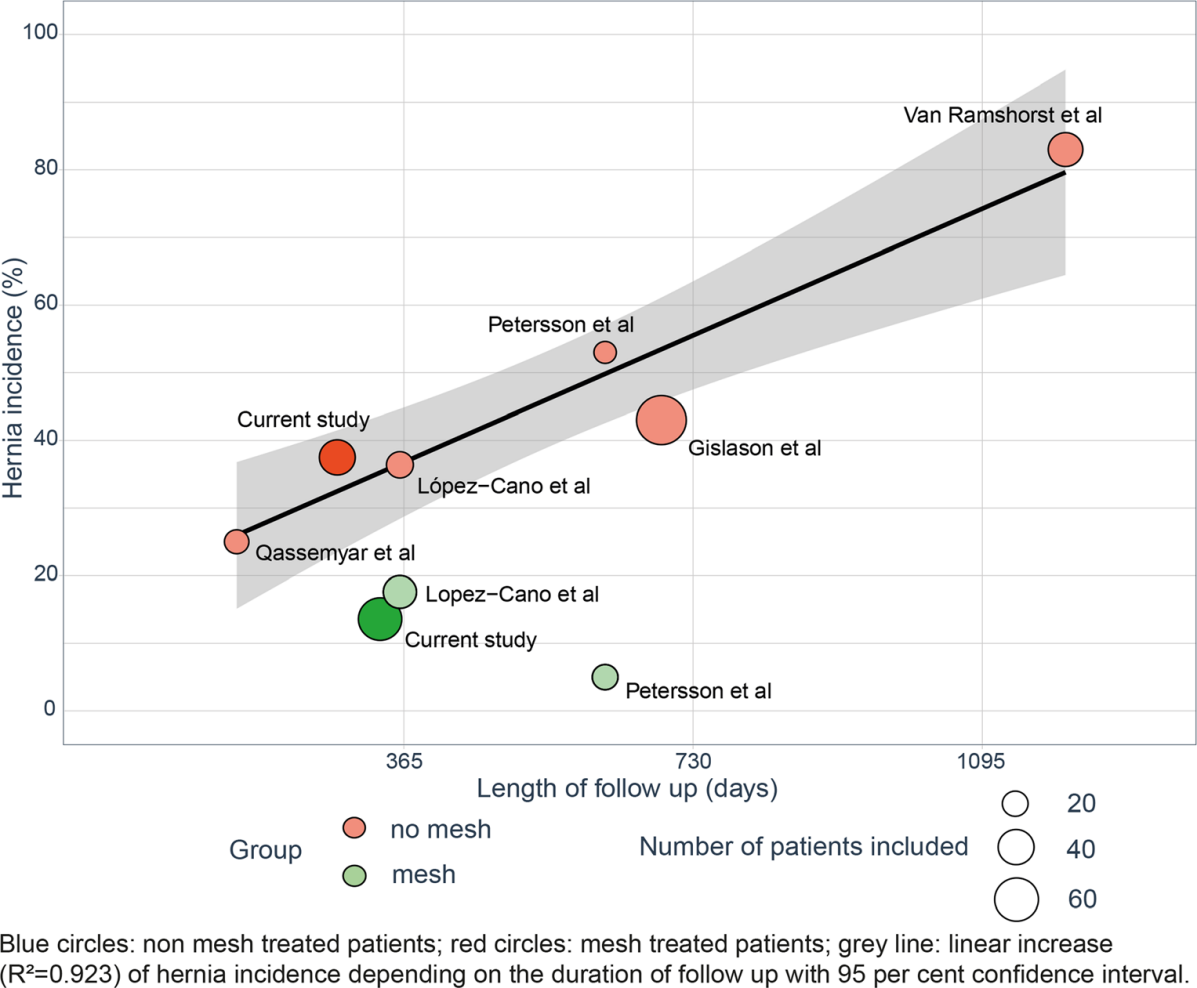
Hernia incidence in patients with fascial dehiscence in published series. Blue circles: non mesh-treated patients; red circles: mesh-treated patients; grey line: linear increase (*R*^2^ = 0.923) of hernia incidence depending on the duration of follow-up with 95% confidence interval

Next, incidence of incisional hernia was compared to published series reporting outcomes with and without non-absorbable meshes in patients with FD (Fig. 2, Tables S2 and S3 (supporting information)).^5^,^6^,^20^–^22^ There is a linear increase (*R*^2^ = 0.923) in incidence of incisional hernia over time without mesh implantation. Pooled odds ratio for incisional hernia formation after mesh implantation was 0.27 (95% CI 0.09–0.76, *P* = 0.013) compared to no mesh (Supplementary fig. S1).^6^,^20^

### Morbidity Related to Mesh Material

**Fig. 3:**
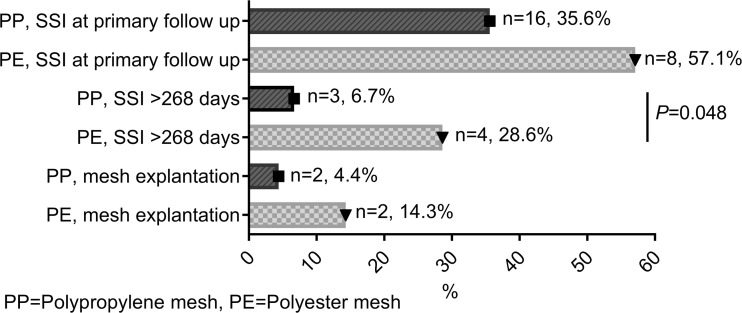
Surgical site infection (SSI). PP polypropylene mesh, PE polyester mesh

Overall, SSI at first follow-up was observed in 24 of 59 patients (40.7%). SSI at first follow-up was found in 16 of 45 (35.6%) patients if a PP mesh and in 8 of 14 (57.1%) patients if a PE mesh was implanted (risk difference − 21.5, 95% CI − 7.9–51.0, *P* = 0.214, Fig. 3). Overall, chronic SSI (duration > 268 days) was found in 7 of 59 (11.9%) patients after mesh implantation. Chronic SSI was found in 3 of 45 (6.7%) with PP mesh and in 4 of 14 (28.6%) with PE mesh. The incidence of chronic SSI with a PE mesh was significantly increased when compared to PP in unadjusted (OR 5.60, 95% CI 1.08–29.11, *P* = 0.040) and adjusted logistic regression analysis (OR 8.69, 95% CI 1.30–58.05, *P* = 0.026). Partial mesh removal was required in 2 of 45 (4.4%) PP meshes, and in 2 of 14 (14.3%) PE meshes (*P* = 0.236) (Table 5).

**Table 5:** Logistic regression analyses of polyester mesh vs polypropylene mesh in patients with FD

	Unadjusted model		Adjusted model	
	Odds ratio (95% CI)	*P* value	Odds ratio (95% CI)	*P* value
SSI at primary follow-up^a^	2.42 (0.71–8.20)	0.157	2.07 (0.49–8.72)	0.323
Chronic SSI > 268 days^b^	5.60 (1.08–29.11)	0.040	8.69 (1.30–58.05)	0.026

Odds ratios are shown polyester versus polypropylene

^a^Adjusted for BMI, tumor, type of laparotomy (median and transverse)

^b^Adjusted for BMI

**Fig. 4:**
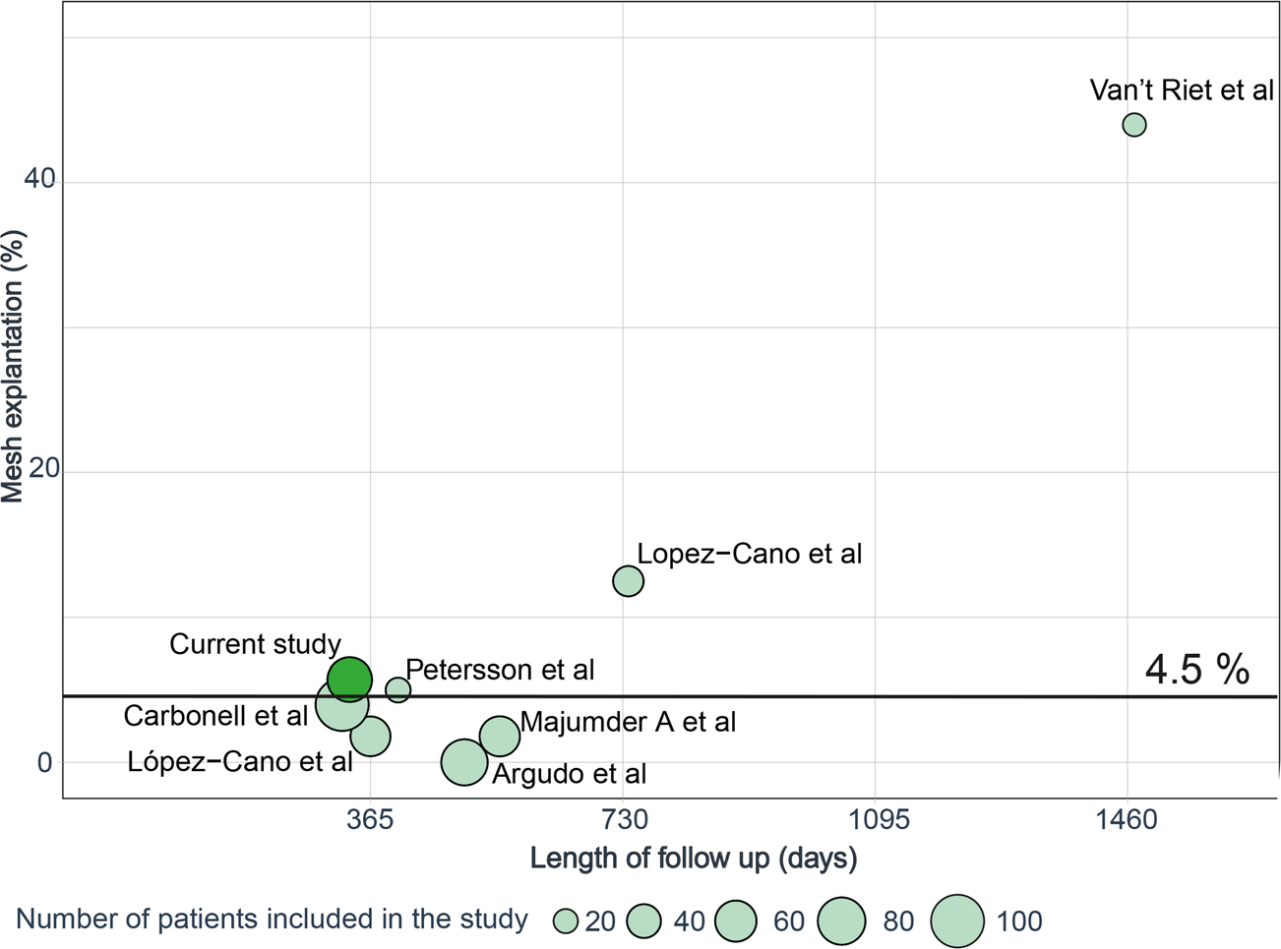
Published series reporting the explantation of non-absorbable meshes in contaminated abdomen

Median percentage of non-absorbable mesh explantation in published series is 4.5 (1.8–10.8) (Fig. 4).^6^,^10^,^12^,^23^–^25^

## Discussion

In the current study, implantation of synthetic, non-absorbable, dual-layered PP mesh was associated with decreased incisional hernia and a low rate of mesh-related morbidity in patients with FD. An important observation is that PE meshes are associated with an increased risk of chronic SSI when compared to PP meshes.

Reported incidence of hernia in patients with FD without mesh implantation ranges from 25 to 83%. Regression analysis of published series addressing the rate of incisional hernia after FD show a linear increase in hernia incidence as shown in Fig. 2a (Supplementing material, Table S2).^5^,^6^,^20^–^22^ Such data highlight the importance of addressing abdominal wall reinforcement in this high risk population. The current study reveals that mesh implantation during repair for FD seems to be an effective therapeutic approach. Recurrence was mainly observed at the mesh borders, which underlines the importance of an adequate surgical technique aiming to sufficiently overlap the incision.

Putative disadvantages of mesh implantation include any type of SSI and intestinal fistula. The current study, however, shows that the incidence of these risks is not increased in patients after mesh implantation compared to controls without meshes from the same institution as well from published series.^26^,^27^ SSI after synthetic mesh implantation in contaminated abdominal cavity have been reported to occur in between 22.8 and 77% of patients (Supplementing material, Table S3).^6^,^10^,^12^,^23^–^25^ The wide variability in reported occurrence of SSI is the consequence of the heterogeneity because of varying inclusion criteria, mesh types, and localization. Exceptional high rates of wound complications of 77% with 17% of enterocutaneous fistulae seem to be the consequence of the use of non-dual-layered meshes.^10^ Such findings underline the relevance of visceral protection that are allowed in part by the use of dual-layered meshes. Thus, fistula formation and mesh explantation were very rare in the current series, which is consistent with other recent publications (Fig. 4).^6^,^10^,^12^,^23^–^25^

The present study reveals that not just visceral protection but also mesh material is of major importance for the success of intraperitoneal mesh implantation. Consistent with our data, Leber et al. already reported in 1998 that PE meshes are associated with a higher incidence of SSI, fistula formation, hernia recurrences, and overall complications compared to PP meshes.^28^ This observation is also supported by experimental data that show significant reduced numbers of colony forming units per gram tissue in PP meshes compared to PE meshes.^15^ This observation was made before the introduction of dual-layered meshes. The present study confirms these results with dual-layered PP that was superior to PE in contaminated abdomen with an OR of 8.69 for chronic SSI using PE-based meshes.

Biologic meshes are a potential alternative to synthetic meshes for the use in FD. However, a recent meta-analysis did not show a benefit for biologic meshes in terms of SSI or hernia recurrence in potentially contaminated hernias.^29^ Conversely, biologic mesh repair is associated with considerable higher rate of surgical site complications and hernia recurrence in *contaminated* hernia repairs compared to synthetic meshes.^12^,^29^ Therefore, the costs of biologic meshes in these settings most likely do not outweigh their benefit. Such hypothesis, however, needs clarification in a prospective controlled trial.

The limitation of this study is its retrospective design and the fact that a historic control was necessary. However, the most important outcome parameters (hernia, fistula, mortality) between groups did not change over the studied period compared to published analyses.^5^,^6^,^20^–^22^ Due to the fact that this is a retrospective study, unlike for symptomatic hernia, asymptomatic hernia might have been missed in our patient’ population. Additionally, the comparison to published series is limited by the quality of the included studies. Given the high rate of success with this approach a randomized trial would be difficult to be accepted by the treating surgeons. Therefore, larger multi-institutional cohorts may allow to further support the current findings.

## Conclusion

The current study shows that implantation of a dual-layered PP but not dual-layered PE meshes in patients with FD decreases incisional hernia with a low rate of mesh-related morbidity.

## Electronic Supplementary Material

Supplementary Fig. S1 (PNG 82 kb)

High resolution image (TIF 10934 kb)

ESM 1 (DOCX 35 kb)
