# Supplementary figures and images for: Prophylactic, Synthetic Intraperitoneal Mesh Versus No Mesh Implantation in Patients with Fascial Dehiscence
Source: J Gastrointest Surg. 2018 Jul 23;22(12):2158–66. doi: 10.1007/s11605-018-3873-z (PMC6244924; doi:10.1007/s11605-018-3873-z)

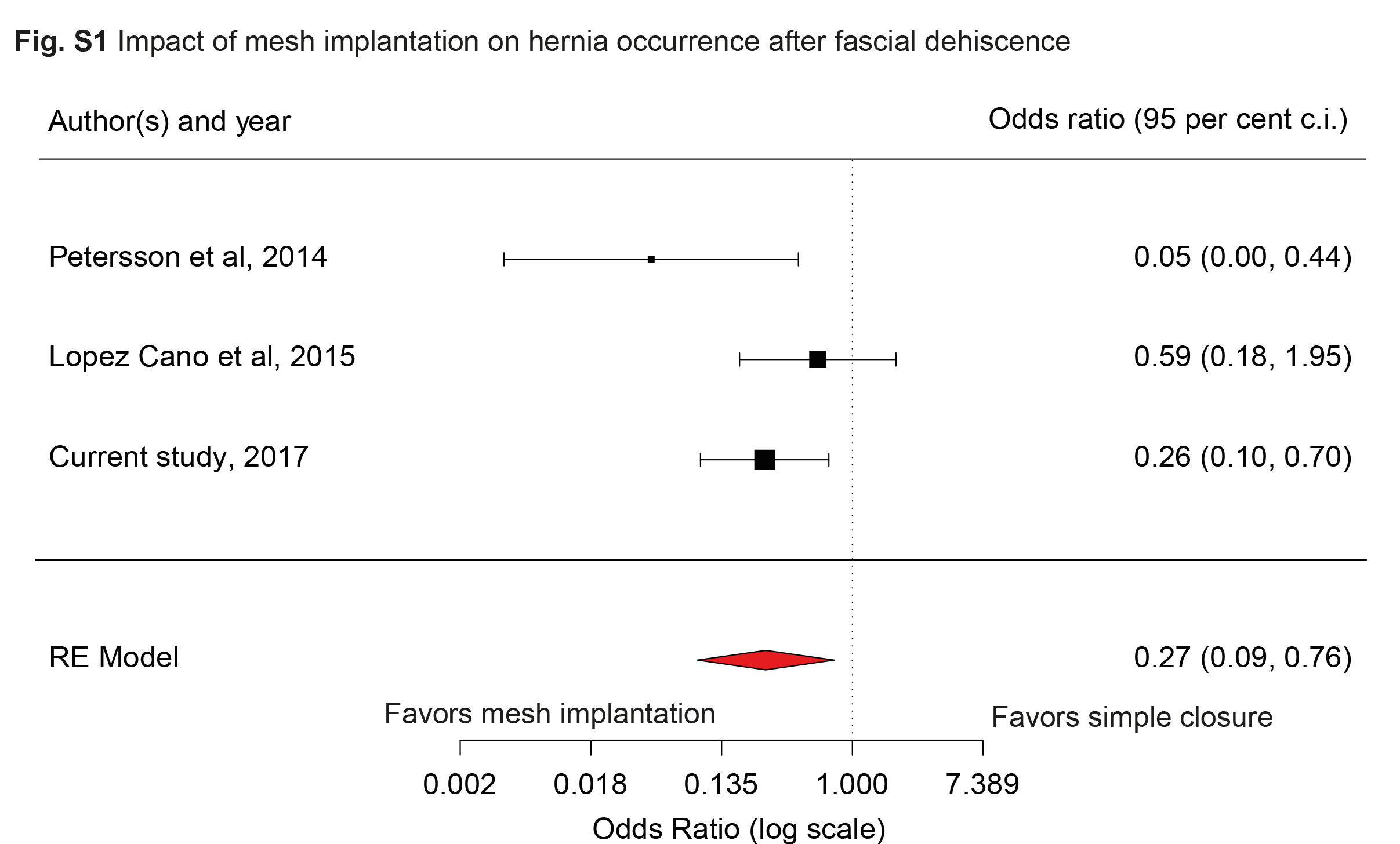

Supplement: Supplementary file 1 — (PNG 82 kb) [file 11605_2018_3873_Fig5_ESM.png]

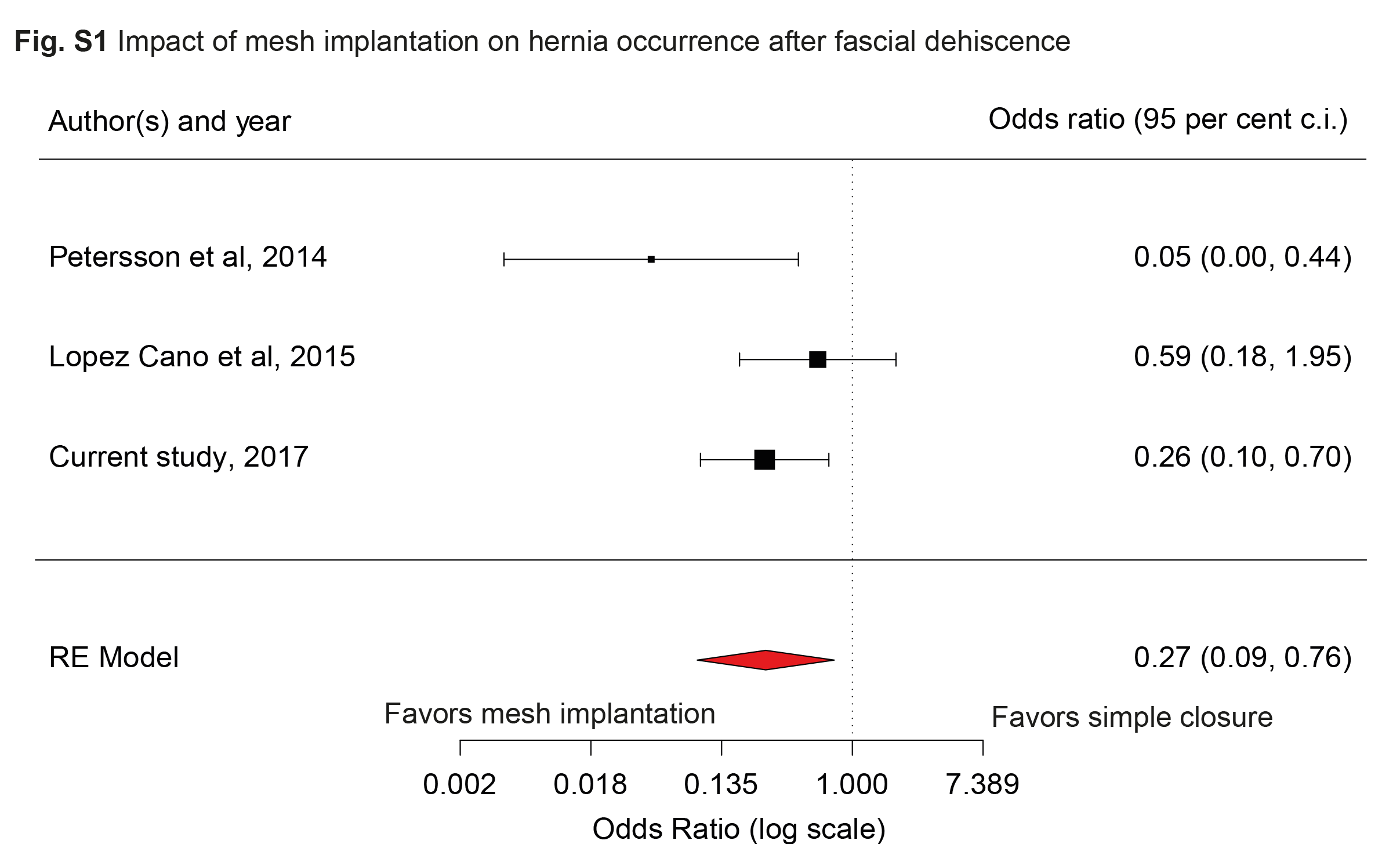

Supplement: Supplementary file 2 — High resolution image (TIF 10934 kb) [file 11605_2018_3873_MOESM1_ESM.tif]
