# Supplementary material for: Prophylactic, Synthetic Intraperitoneal Mesh Versus No Mesh Implantation in Patients with Fascial Dehiscence
Source: J Gastrointest Surg. 2018 Jul 23;22(12):2158–66. doi: 10.1007/s11605-018-3873-z (PMC6244924; doi:10.1007/s11605-018-3873-z)
Supplement: Supplementary file 3 — (DOCX 35 kb) [file 11605_2018_3873_MOESM2_ESM.docx]

**Supporting information**

**Table S1** Patients with non-adherence to protocol after 2006

| Year | Primary operation | Concomitant complication | Secondary operation | Reason for no mesh in fascial dehiscence | In-hospital mortality |
| --- | --- | --- | --- | --- | --- |
| 2006 | Major lung surgery because of bronchial carcinoma | Small intestinal leakage of unknown reason and cholecystitis | Sewing of small intestine and cholecystectomy | No mesh due to severe contamination and underlying disease | No |
| 2007 | Fournier gangrene because of perianal abscess | Complications due to Fournier Gangrene | Debridement of fascia | No mesh due to underlying disease | Yes |
| 2007 | Left sided hemicolectomy due to colonic tumor | Anastomotic leakage | Stoma, severe contamination (Mannheim Peritonitis Index 19) | Conservative therapy | No |
| 2007 | Resection of rectal tumor | Anastomotic leakage | Hartmann procedure | Severe contamination and potential of revisional surgery | No |
| 2008 | Perforated diverticulitis of sigmoid colon | Anastomotic leakage | Hartmann procedure | Fistula of small intestine | No |
| 2008 | Laparoscopic adnexectomy | Sigmoid perforation | Hartmann procedure | No mesh due to contamination (Mannheim Peritonitis index 26) | No |
| 2009 | Hartmann procedure because of sepsis due to sigmoidovesical fistula | Septic shock | -- | Conservative therapy | Yes |
| 2014 | Cholecystectomy because of cholecystitis | Fascial dehiscence | Simple closure | Already implanted mesh in 2009 | No |
| 2014 | Gastric bypass | Fascial dehiscence | Simple closure | Surgeons decision | No |
| 2015 | Extended left-sided hemihepatectomy | Partial liver necrosis | Necrosectomy | No mesh due to underlying disease and the potential of revisional surgery | Yes |

**Table S2** Hernia incidence of non-mesh treated patients with FD

| References | Year | Study type | *n* | Technique of closure | SSI  (%) | Mortality  (%) | Hernia  (%) | Follow-up |
| --- | --- | --- | --- | --- | --- | --- | --- | --- |
| Gislason et al [21] | 1999 | Retrospective | 78 | Sutures, Retention sutures | n/a | 14 | 43 | 23 months |
| Qassemyar et al [22] | 2011 | Prospective | 18 | Tension sutures | n/a | 11 | 25 | 154 days |
| Van Ramshorst et al [5] | 2013 | Retrospective | 37 | Conservative / suture / mesh | n/a | 16 | 83 | 40 months |
| Petersson et al [6] | 2014 | Retrospective | 15 | Suture | 28 | 5 | 53 | 619 days |
| López-Cano et al [**20**] | 2015 | Retrospective | 35 | Suture | 20 | 31.4 | 36.4 | >12 months |

**Table S3** Mesh related morbidity after implantation of synthetic, non-absorbable mesh in contaminated abdomen

| Reference | Year | Type of mesh | n | Mesh location | Enteric fistula (%) | SSI  (%) | Mesh explantation  (%) | Mortality  (%) | Follow-up |
| --- | --- | --- | --- | --- | --- | --- | --- | --- | --- |
| Van’t Riet et al[10]* | 2007 | PP / PE | 18 | Inlay | 17 | 77 | 44.0 | 33.0 | 49 months |
| Petersson et al[6]* | 2014 | PP | 21 | Sublay | n/a | 71 | 5.0 | 6.0 | 405 days |
| López-Cano et al[**20**]* | 2015 | PP | 56 | Onlay | n/a | 29.1 | 1.8 | 14.3 | >12 months |
| Carbonell et al[23]† | 2013 | PP | 100 | 94% Sublay, 5% IPOM, 1% Onlay | 1 | 31 | 4.0 | n/a | 10.8 months |
| Argudo et al[24]† | 2014 | PP | 76 | Onlay | n/a | 26 | 0 | 18.0 | 16.7 months |
| Majumder A et al[12]† | 2016 | PP / PE | 57 | Sublay | n/a | 22.8 | 1.8 | n/a | 18.4 months |
| Lopez-Cano et al[25]† | 2017 | n/a | 32 | Sublay/ Onlay | 6.2 | 28.1 | 12.5 | n/a | 24.6 months |

*Patients with fascial dehiscence and mesh implantation. †Patients after mesh implantation in contaminated abdomen. PP, polypropylene mesh; PE, polyester mesh.
